# The effect of breed-specific dog legislation on hospital treated dog bites in Odense, Denmark—A time series intervention study

**DOI:** 10.1371/journal.pone.0208393

**Published:** 2018-12-26

**Authors:** Finn Nilson, John Damsager, Jens Lauritsen, Carl Bonander

**Affiliations:** 1 Department of Environmental and Life Sciences, Karlstad University, Karlstad, Sweden; 2 Centre for Public Safety, Karlstad University, Karlstad, Sweden; 3 Accident Analysis Group, Department of Orthopedics and Traumatology, Odense University Hospital, Odense, Denmark; 4 Department of Clinical Medicine, University of Southern Denmark, Odense, Denmark; 5 Institute of Medicine, Health Metrics Unit, University of Gothenburg, Gothenburg, Sweden; Medical University Graz, AUSTRIA

## Abstract

As dog bite injuries are a considerable problem in modern society, in order to reduce such injuries, breed-specific legislation has been introduced in a number of countries. Whilst many studies have shown a lack of effect with such legislation, the commonly used methodology is known to be flawed. Therefore, the aim of this study is to investigate the effect of the Danish breed-specific legislation on the number of dog bite injuries using more credible methods. A time series intervention method was used on a detailed dataset from Odense University Hospital, Denmark, regarding dog bite injuries presented to the emergency department. The results indicate that banning certain breeds has a highly limited effect on the overall levels of dog bite injuries, and that an enforcement of the usage of muzzle and leash in public places for these breeds also has a limited effect. Despite using more credible and sound methods, this study supports previous studies showing that breed-specific legislation seems to have no effect on dog bite injuries. In order to minimise dog bite injuries in the future, it would seem that other interventions or non-breed-specific legislation should be considered as the primary option.

## Introduction

Dog bites are a considerable problem in modern society. On average, the risk of receiving a dog bite severe enough to require medical attention is 3-4/1000 population per year [1], meaning that globally, dog bites are amongst the top 12 causes of nonfatal injuries [2]. According to epidemiological studies, the risk factors of dog bite injuries can be subdivided in to person-related, dog-related and environmental factors. With regards to victims, a number of studies have shown that children are substantially more injured than adults and that male children are more often injured than female children [1, 3, 4]. Environmentally, bites can occur both in the public and the private. When it comes to dog-related factors, the risk also appears to vary by breed. Due simply to large populations, popular breeds are often represented in the data. However, there are also other breeds that seem to be overrepresented in the data per population. More specifically, studies have shown that pit-bull type dogs, German Shephard type dogs and cross-breeds seem to injure people more than other breeds [1, 3, 5].

In an attempt to combat this problem, judicial regulations have been suggested and implemented in a number of countries [6–9]. Specifically, two different approaches have been used; breed specific legislation and non-breed specific legislation. Whilst breed-specific legislation is focused on the banning of ownership and breeding of particular breeds that have been identified as dangerous, non-breed-specific legislation is focused on promoting responsible dog ownership. Commonly, breed-specific legislation is the preferred strategy, likely due to the expectation of an abrupt effect on the number of dog bites given that the assumption is that certain breeds account for a majority of dog bite injuries. Although breed-specific legislation differs between countries, studies from Europe, Australia and North America have generally shown that the evidence of an effect is weak [6, 9–11], although some studies claim to have found an effect [12, 13]. A general problem, however, with the studies on breed-specific legislation that we have found, is the chosen methodology. Specifically, a before/after-design has most often been used, which is based on the assumption that there are no time trends in the data, and that the effect can be identified using a simple difference in pre-post means. However, there are many reasons to suspect that these assumptions are violated. The most obvious is that when estimating the effects of safety legislation, the issue of secular trends must be taken into account, especially since injury data tends to trend downwards [14]. Furthermore, we expect that the effects might vary over time considering that certain breeds are phased out gradually after being banned, which would likely produce an effect with a complex functional form that cannot be capture by a simple comparison of means.

In this study, we focus on the case of a breed-specific legislation introduced on June 1^st^ 2010 in Denmark (*Hundeloven*, *§1a and §1b*), banning the breeding, import and new ownership of thirteen breeds, specifically identified as dangerous. For two of these breeds (Pitbull terrier and Tosa Inu), all existing dogs were even ordered to be euthanized. For the remaining eleven (American Staffordshire Terrier, Fila Brasiliero, Dogo Argentino, American Bulldog, Boerboel, Kangal, Central Asian Ovtcharka, Caucasian Ovtcharka, Tornjak and Sarplaninac), an intermittent law was imposed on existing dogs meaning that these were forced to wear a muzzle and be on a leash in public places at all times. Hence, if the law is effective, we expect the effect to take on different shapes over time depending on location. Given the gradual phase-out of eleven of the thirteen breeds, we expect that the average effect of the law will gradually increase over time. However, since all dogs that are covered by the law are required to be muzzled in public places, there should also be an instant effect in public spaces that goes beyond this average effect. To test these hypotheses empirically, we apply a novel time series intervention method to a detailed dataset from Odense University Hospital, where we explore the effects of the breed-specific legislation on dog bite injuries in both private and public spaces with minimal modelling assumptions.

## Materials and methods

### Material

The data for this study was collected from Odense University Hospital in Odense, Denmark. Odense is the third largest city in Denmark with 188,000 inhabitants (5% of the Danish population) and has one hospital with an emergency department. All injuries presented to the emergency department are routinely registered according to the NOMESCO cause of injury classification principles [15] with regards to cause, injury type and background factors with regards to the individual. This data is then compiled and owned by the Accident Analysis Group at Odense Hospital. Included in this study were all bog bite cases presented to the emergency department between January 1^st^ 2002 and June 31^st^ 2015.

The Accident Analysis Group at Odense Hospital deemed that no ethical approval was needed for this study given that no information regarding the victims was included. Rather, merely data regarding the month of the emergency department visit and whether the dog bite occurred in a public or private space were included.

### Data analysis

We began by performing a “naïve” before-after analysis by stratifying the data by intervention status and location, using Poisson regression to quantify rate differences and rate ratios. Recognizing that intervention effect estimates stemming from this estimator are often biased by time trends (which are often prevalent in accident and injury data), we then moved on to estimating a set of time series intervention ARIMA models using log-transformed versions of the outcome variables. Since the intervention takes place at (roughly) the half-year mark, we stratified the data into two time-series (one per location of the injury) where each observation represents a 6-month period. To avoid making any strict assumptions regarding the shape of the intervention effect in the model, we performed out-of-sample forecasting using the ARIMA(*p*,*d*,*q*) model that provided the fit in the pre-intervention period, where *p* represents the autocorrelation (AR) order, *d* the trend differencing parameter and *q* the moving average (MA) order (see e.g. [16] for more details on ARIMA models). We used plots of the autocorrelation (ACF) and partial autocorrelation function (PACF) as a guide in choosing the best model for each outcome variable, which showed that AR(1) or MA(1) was usually sufficient to deal with the autocorrelation in the data, and ultimately selected the model that minimized the root mean prediction error (RMSE) in the pre-intervention period (out of the candidate models; see Table A in S1 File for details).

To quantify the estimated intervention effect along with uncertainty intervals, we then performed a Monte Carlo simulation study assuming a log-normal distribution around the predicted values (with standard errors obtained from the model predictions) and a Poisson distribution around the observed values, which is the most common assumption for the variance function in aggregate count data [17]. Since the Monte Carlo method relies on random number generation, the coefficients will naturally vary per completed simulation, but according to the law of large numbers, they will converge at the population parameter as the number of iterations approaches infinity (for more details, see e.g. [18]). The simulations were therefore iterated 10000 times in order to obtain a high coefficient stability.

## Results

A total of 2622 dog bite injuries were recorded throughout the study period (1^st^ January 2002 to June 31st 2015), 1748 of which occurred private spaces and 874 in public spaces (Table 1). As can be seen in Table 1, the naïve before-after analysis suggests that the ban significantly reduced the number of dog bite injuries in Odense by 15%. However, this result is specific to private spaces, which by the nature of the new law should be less affected than dog bites occurring in public spaces.

**Table 1:** Dog bite injuries in patients presenting at Odense University Hospital by period (before and after the law change) and location.

*Location*:	All	Private spaces	Public spaces
	**A. Number of dog bite injuries**		
**Before**	1748	1269	480
**After**	874	610	264
	**B. Average 6-month rate**		
**Before**	102.82	74.65	28.24
	(98.06, 107.70)	(70.54, 78.75)	(25.71, 30.76)
**After**	87.40	61.00	26.40
	(81.61, 93.19)	(56.16, 65.84)	(23.22, 29.58)
	**C. Naïve before-after analysis**		
**Rate difference (after-before)**	-15.42	-13.65	-1.84
	(-23.02, -7.94)	(-20.00, -7.30)	(-5.90, 2.23)
**Rate ratio (after/before)**	.85	.82	.94
	(.78, .92)	(.74, .90)	(.80, 1.09)

95% confidence intervals are estimated assuming a Poisson error distribution.

**Fig 1:**
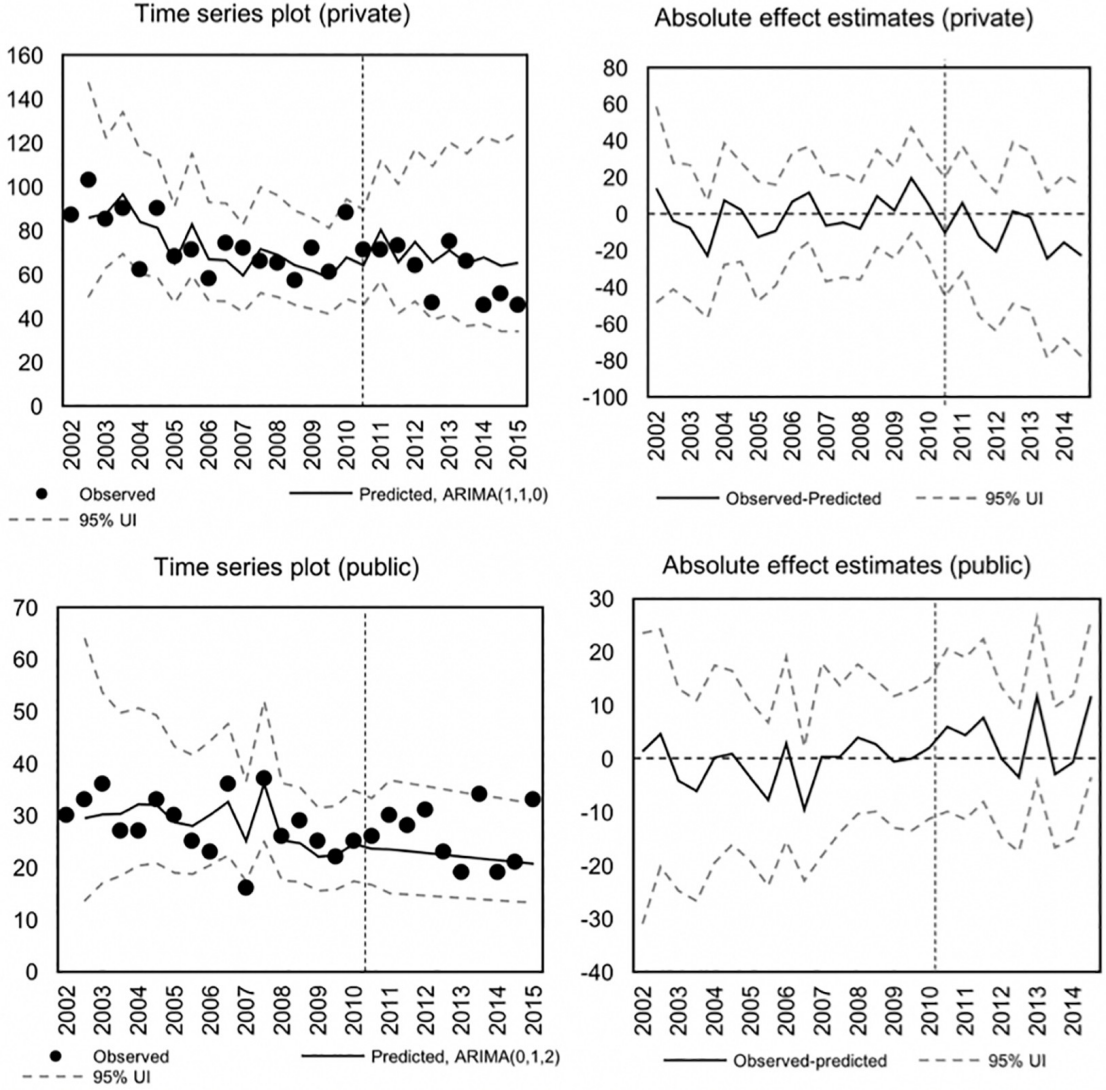
Time series plots over the number of dog bite injuries per 6-month period and location, with predicted values and estimated effects. (UI = Uncertainty interval).

After testing a range of ARIMA(*p*,*d*,*q*) specifications, we found that the optimal model for the natural log of dog bite injuries occurring in private spaces was an ARIMA(1,1,0) model, while an ARIMA(0,1,2) model provided a better fit for injuries in public spaces (see Table A in S1 File). Autocorrelation plots are available in Figs A and B in S1 File, where we can see that there is no evidence of residual autocorrelation in the final models. The fitted and observed values are presented in the left column of Fig 1, and the estimated effects based on the Monte Carlo simulations are shown in the right column. We found no statistically significant evidence after accounting for the secular trends in data. More specifically, the out-of-sample predictions from the time series models do not differ significantly (at the 0.05-level) from the observed values in the post-period, for any of the outcome variables.

Quantifying the average effect estimates in the post-period, we found that the average effect estimate for dog bites in private spaces was -19.16 (95% UI: -109.9, 48.59) in terms of annualized rate differences, or -7.53% if expressed as a percentage change (95% UI: -49.18, 55.64). The corresponding estimates for dog bites in public spaces was +7.14 (95% UI: -22.63, 34.46) and +21.63 percent (95% UI: -36.46, 105.12). Hence, the available evidence does not suggest that the law has had an impact on injuries due to dog bites in Odense, Denmark.

### Discussion

Despite using more advanced methods, the results from this study seem to confirm the conclusions from previous studies that show that breed-specific legislation is ineffective in reducing the number of patients with dog bites presented to medical services [6, 9, 10, 19, 20]. It would seem, therefore, that banning certain breeds has a highly limited effect on the overall levels of dog bite injuries, and that enforcement of the usage of muzzle and leash in public places for these breeds has a limited effect.

From a theoretical perspective, the lack of effect could be seen as surprising given that the banned breeds have a reputation of being aggressive. However, although, as mentioned previously, some breeds are over represented in dog bite statistics [1, 3], there is a lack of evidence demonstrating a higher rate of aggression in certain canine breeds [20]. Interestingly, German Shepherds, which have been identified as a high-risk breed, were not banned, which also calls into question the selection process of dangerous breeds. Most importantly, however, despite certain breeds having a proportionally higher risk than others, the dogs most often inflicting dog bite injuries are the popular breeds, purely due to the number of individuals. Also, by merely banning certain, less popular breeds, the potential reduction is very low [21]. Therefore, by disregarding the most popular breeds it will be unlikely that any law will affect the number of dog bites to any larger extent. Similarly, whilst the banning of certain breeds seems logical on paper, the issue of mixed breeds is complicated, especially given that mixed breeds are also identified as high-risk. How mixed breeds have been handled in the Danish legislation is unclear given that these are not mentioned in the specific paragraphs.

A further misinterpretation with regards to the Danish breed-specific legislation seems to be with regards as to where dog bites occur. Given the regulating of so-called dangerous breeds in public spaces, but ignoring private spaces, the legislators seem to have thought that most dog bites are in the public sphere, inflicted by dogs towards strangers. However, the results in this study clearly shows that dog bites are exceedingly more common in private spaces, an aspect that has also been evident in previous studies [1].

As was mentioned in the background, in an effort to minimise dog bites to humans, three aspects can be targeted; dog-related, environment-related and victim/owner-related. The Danish breed-specific legislation attempted to target both dog-related and environment-related, though not victim/owner-related. Previous studies have shown that between 20 and 33% of all attacks are on family members [1, 7, 22]. Given this aspect, it is also unsurprising that the breed-specific legislation seems to have been ineffective. Rather than breed-specific legislation, non-breed-specific legislation, for example obligatory de-sexing [23] or education programmes [24], would have a greater chance of being effective in reducing dog bites. This is further highlighted by the fact that previous studies have shown that the most over-represented breeds vary over time, depending upon trends [1], indicating that the risk of dog bites is not connected to the breed, but rather the owner, an aspect untouched by breed-specific legislation.

### Strengths and limitations

Firstly, a potential limitation to our study is a low statistical power to detect small effects. As a reference point, sample size calculations for the Poisson pre/post-test suggest that 20 and 54 years of observation would be required to detect a statistically significant effect of -10% in public spaces and private spaces, respectively, given the baseline injury rates. This is considerably longer than our 17.5-year time frame, at least for private spaces. On the other hand, an effect size of -20% would require two (for public spaces) and 13 years of observation (for private spaces) and -30% only two and five years. However, these numbers only serve as an indication given that our main analysis is based on ARIMA models, and assume that there are no secular trends in the data.

Secondly, we based our inferences solely on observed data in one intervention population without access to concurrent controls. While we were able to convincingly account for time trends as observed in the pre-intervention period, our projections relied solely on the evolution of dog bite injuries as observed in the pre-period. This is an inherently weaker design for causal inference than e.g. difference-in-differences or synthetic control methods [25, 26], which could not be used in this case since they require control data from non-intervention populations. Still, our use of ARIMA modelling techniques and forecasting enabled us to make fewer modelling assumptions regarding the functional form of the intervention effect than in the case of segmented regression analysis in interrupted time series analyses [14, 27]. This aspect, together with the Monte Carlo simulations, allowed us to produce year-by-year counterfactual effect estimates, which (to our knowledge), is a unique application in time series intervention analysis. Additional strengths lie in the quality of the dataset, which contains the most detailed and reliable injury data over an extended time period in Denmark. The qualities in the dataset allowed us to identify dog bite injuries and stratify the outcome variable by location, which not only enabled the study to begin with, but also allowed us to test for differential effects between private and public spaces. However, no data is collected with regards to breed when dog bite victims are presented. Whilst this would be beneficial in a preventative perspective, previous studies have indicated that such information is often flawed [28, 29].

Moving on to the external validity, it should be noted that the limited geographical coverage of the data may be an issue, especially since the sampling of patients presenting at Odense University Hospital is a non-random sample of the Danish population (by definition). Although the Odense area covers urban and rural areas, and a varied social background population comparable to the rest of Denmark, we cannot be certain whether or not the results are generalizable to other parts of Denmark. However, this should not be a large problem if the distribution of dangerous dog breeds (as defined by the breed-specific legislation) is similar in other regions as well. As we have not had access to data on dog ownership in Odense or Denmark, this has not been possible to control for. Still, our results are consistent with most previous studies on the topic of breed-specific legislation conducted in other countries, which lends some additional credibility to our estimates. An additional limitation is the scope of the post-period, which at present is only sufficient for studying short run effects. Since some components of the legislation may have a delayed effect that might not yet be fully realized after four years, it is important to return to this case in the future.

## Conclusions

According to the results in this study, no effect of the legislation can be seen on the total number of dog bites, therefore supporting previous studies in other countries that have also shown a lack of evidence for breed-specific legislation. Importantly, compared to other studies, this study can show a lack of evidence using more robust methods, therefore further highlighting that future legislation in this area should be prioritized on non-breed-specific legislation in order to reduce the number and risk of dog bites.

## Supporting information

S1 File Table A. Root mean squared error from different ARIMA(p,d,q) specifications.Fig A. Plot of the autocorrelation (ACF) and partial autocorrelation (PACF) functions for the optimal model for dog bites in private spaces (ARIMA(1,1,0)).Fig B. Plot of the autocorrelation (ACF) and partial autocorrelation (PACF) functions for the optimal model for dog bites in public spaces (ARIMA(0,1,2)).
(DOCX)

S1 Dataset (XLSX)
