# Supplementary material for: The effect of breed-specific dog legislation on hospital treated dog bites in Odense, Denmark—A time series intervention study
Source: PLoS One. 2018 Dec 26;13(12):e0208393. doi: 10.1371/journal.pone.0208393 (PMC6306151; doi:10.1371/journal.pone.0208393)
Supplement: S1 File — Table A. Root mean squared error from different ARIMA(p,d,q) specifications.Fig A. Plot of the autocorrelation (ACF) and partial autocorrelation (PACF) functions for the optimal model for dog bites in private spaces (ARIMA(1,1,0)).Fig B. Plot of the autocorrelation (ACF) and partial autocorrelation (PACF) functions for the optimal model for dog bites in public spaces (ARIMA(0,1,2)). (DOCX) [file pone.0208393.s001.docx]

# **S1 File**

Table A.

| Model specification | Private spaces | Public spaces |
| --- | --- | --- |
| ARIMA(0,0,0) | .176 | .213 |
| ARIMA(0,1,0) | .226 | .360 |
| ARIMA(0,2,0) | .413 | .693 |
| ARIMA(1,1,0) | .159* | .249 |
| ARIMA(2,1,0) | .164 | .221 |
| ARIMA(0,1,1) | .176 | .224 |
| ARIMA(0,1,2) | .175 | .183* |
| ARIMA(1,1,1) | .163 | .190 |

^Notes: *Model that minimizes the RMSE, used in the main results.^


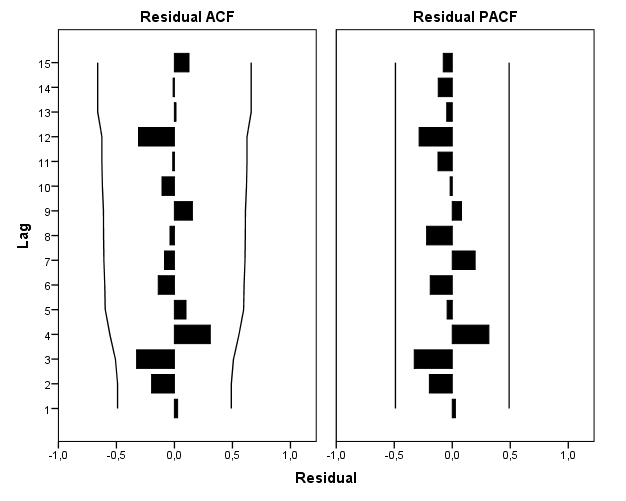


Fig A.


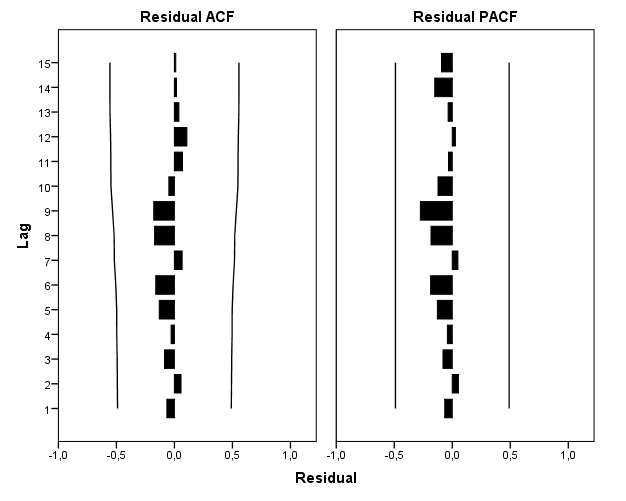


Fig B.
